# Review: application and opportunities for machine learning and artificial intelligence in preclinical immunogenicity risk assessment

**DOI:** 10.3389/fimmu.2026.1720928

**Published:** 2026-05-28

**Authors:** Timothy Paul Hickling, Morten Nielsen, Pieter Meysman, Rachel H. Rose, Olga Obrezanova

**Affiliations:** 1 Roche Pharma Research and Early Development, Roche Products Ltd., Welwyn Garden City, United Kingdom; 2 Quasor Ltd, Loughborough, United Kingdom; 3 Section for Bioinformatics, Department of Health Technology, Technical University of Denmark, Lyngby, Denmark; 4 Adrem Data Lab, Department of Computer Science, University of Antwerp, Antwerp, Belgium; 5 Certara Predictive Technologies, Applied BioSimulation, Sheffield, United Kingdom; 6 Biologics Engineering, Oncology R&D, AstraZeneca, Cambridge, United Kingdom

**Keywords:** artificial intelligence, biologic therapies, immunogenicity, machine learning, major histocompatibility complex (MHC), quantitative systems pharmacology (QSP)

## Abstract

The unwanted immune response to Biologic Therapies can result in anti-drug antibodies that complicate clinical development and may adversely affect patient outcomes. At present, prediction of the impact of this immunogenicity before starting clinical trials is impossible, due to the complexity of the immune system and the multiple factors that contribute to the risk of immunogenicity. Advances in computational methods and power will enable improvements in prediction of immunogenicity. A workshop at EMBL-EBI brought together industry experts and academics to reflect on the contributions of artificial intelligence (AI) and machine learning (ML) to immunogenicity prediction, to review current practices across industry, and to look to future opportunities for applying AI technologies. This review was inspired by the topics and discussions presented at the workshop. Machine learning has been employed for immunogenicity prediction for more than 20 years. Specifically, the prediction of peptides bound by the Major Histocompatibility Complex (MHC) Class II molecule has helped to identify potential T cell epitopes, which can be used for selecting candidates with low immunogenicity risk, informing protein engineering for reducing risk, or informing risk assessments and immunomonitoring during clinical trials. Application of ML algorithms in data rich disease areas such as haemophilia is informative for clinical decision making. ML and other AI techniques require large data sets which have been acquired through consistent methods. A challenge for immunogenicity prediction is the harmonization of preclinical risk assessment assays and the clinical measurements of anti-drug antibodies. With imperfect data, quantitative systems pharmacology (QSP) modelling has been applied to link together the immune system with observations of risk factors, with simulations of clinical trials providing a perspective on the immunogenicity risk. Industry workflows are aligned on application of tools and recognise gaps that need to be filled with additional data and assays. Further innovation in modalities requires extension of the risk assessment paradigm and will demand further innovation in immunogenicity prediction approaches. Finally, we address opportunities for AI/ML to solve key questions and reflect on the challenges in validating the predictive capabilities of new models.

## Introduction

Biological therapies provide treatments for many of the most significant diseases across therapeutic areas such as Oncology, Immunology, Cardiovascular, and Rare diseases. Development of biological therapies face challenges common with other therapies, such as the potency and durability of effect. Furthermore, specific developability issues concerning drug-like properties for biologics are being extensively studied to increase the likelihood of launching a new medicine. A persistent factor in the development of biologics is the unwanted immune response, mostly manifested in terms of anti-drug antibodies (ADAs) that patients may generate to the drug (1). At this time, it is difficult to predict the extent and impact of the immunogenicity of these drugs. The industry standard is to conduct a risk assessment prior to clinical studies (2). The risk assessment includes aspects related to the product, the patients and the way the treatment will be given. Preclinically, several assays exist to understand mechanisms of immunogenicity and preclinical studies may give an insight into potential consequences of any observed immune response (3). During clinical development, appropriate measurement of ADAs using validated assays is a regulatory requirement (4, 5). Monitoring of volunteers and patients during clinical trials is usually performed without intervention. In the rare cases of adverse events related to immunogenicity, clinical mitigations usually involve withdrawal of the drug and, if needed, steroid treatment to overcome hypersensitivity reactions (6). With products where a high risk of immunogenicity occurrence and impact is anticipated, pre-emptive use of steroids can be planned. Post-marketing experience indicates that some therapies may lose their efficacy for substantial numbers of patients due to immune effects (7). There is general agreement that being able to predict these outcomes early in drug discovery and development would result in better long-term treatments for patients.

**Figure 1:**
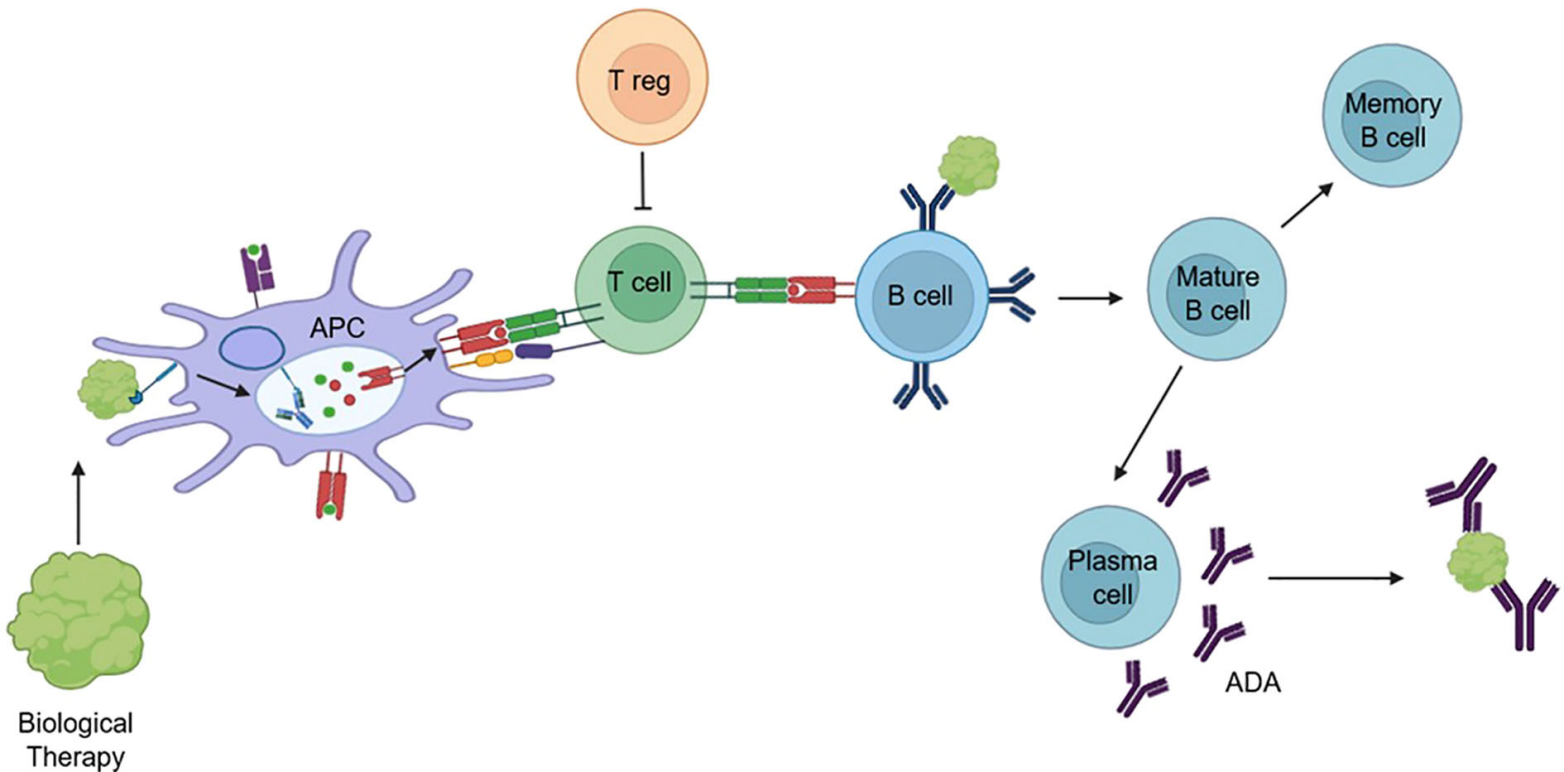
Immune system processes that contribute to the production of anti-drug antibodies. The processes resulting in immunogenicity to biological therapies are like immune responses to vaccines or microbes. In most cases, biologics need to be recognized by both T (CD4) and B cells to generate sufficient ADA to be clinically significant. T cell recognition of biologics relies on uptake, processing and presentation of peptides by antigen presenting cells, together with specific recognition of the HLAII-peptide complex presented through the T cell receptor. B cells bind directly to biologics through the surface expressed BCRs. Presentation of the same peptide from the biologic from a B cell and an APC to a T cell results in maturation of the B cell response, including increased affinity and class switching of the ADA. The mature B cells differentiate into Plasma cells which generate large quantities of secreted antibodies and memory cells, which can respond more easily to future exposure to the same antigen.

The processes of the immune system and the risk factors for immunogenicity are reasonably well understood (**Figure 1**), giving a framework for immunogenicity risk assessment. Across industry, computational and *in vitro* tools for preclinical immunogenicity assessment are routinely applied (1). It is widely accepted that the tools have a limited ability to predict clinical immunogenicity, yet they do provide insight for making decisions for designing and selecting candidates, and for setting risk levels for monitoring immunogenicity during clinical development. With the emergence of new modalities, such as cell therapy and multi-specific antibodies, uncertainties exist regarding the application of the current methodologies (8).

With the development of hundreds of biological therapies, the expectation of predicting unwanted immune responses is increasing. However, the number of variables that contribute to risk is high and the diversity of the immune system across different patients is broad. Immunogenicity prediction is therefore considered a ‘complex’ problem which is likely to need extensive detailed data sets and complex models to solve. Over the last decade progress has been made in a number of areas that may contribute to the solutions. EMBL-EBI brought together industry experts to discuss progress in the field of Preclinical Immunogenicity Assessment of Biologics, highlighting the application of tools to current risk assessments and looking to which areas need improvements in order to adequately predict immunogenicity for clinical trials. The meeting focused on new developments in AI and computational tools for immunogenicity prediction with a view to improving networking and to explore precompetitive collaborations between industry and academia.

## Current predictive capabilities – *in silico*

### HLA-peptide interactions

A critical selective factor influencing the onset and fate of host adaptive immune responses is antigen presentation by human leukocyte antigen (HLA) molecules (see **Figure 1**), also known as Major Histocompatibility Complex (MHC) molecules. Over the last two decades the journey towards HLA antigen presentation prediction has resulted in highly accurate models. Performance of these methods has been improved both by the increased availability of training data and the development of novel machine learning architectures. A major challenge faced when developing prediction models for HLA antigen presentation is the high polymorphism in HLA molecules, with over twelve thousand Class II alleles identified (9). A key breakthrough addressing this was the development of pan-specific models that allowed extrapolation to uncharacterized molecules from the limited set of HLAs characterized by binding data (10).

Another critical source of improvement was the technological advances in proteomics and mass spectrometry enabling the study of immunopeptidomes, i.e. the set of peptides presented by HLA in a given cell (11, 12). This data is often obtained from primary cells expressing up to twelve different HLAs, and a key challenge associated with the application of such immunopeptidome data is to assign peptides to one or more of these HLAs responsible for the antigen presentation. Moreover, for HLA class II molecules, the bound peptides are most often of length 12–21 with a binding core of nine residues with no fixed location (13). This greatly challenges the specificity learning task, since peptides must first be aligned to a common binding core before the binding rules can be inferred. Several approaches have been suggested to resolve these challenges including NNAlign, a neural network-based method for sequence motif identification in quantitative peptide data (14, 15), and NNAlign_MA, a machine learning method concurrently performing HLA motif deconvolution and training of pan-specific binding predictors (16).

For HLA class II, applying these approaches to large immunopeptidome data has resulted in several new algorithms including the NetMHCIIpan 4.3 method (17), Graph-pMHC (18), MixMHC2pred (19, 20) and HLAIIPred (21) (**Table 1**). During development of NetMHCIIpan, several critical issues related to the available immunopeptidome data became apparent. Firstly, accurate HLA motif deconvolution revealed a significant contribution of DRB3, 4 and 5 to the total DR immunopeptidome (22). Secondly, the work highlighted the presence of co-immunoprecipitated HLA irrelevant “contaminants”. For HLA class II, these “contaminants” were found to be predominantly located as singletons, i.e. not in nested sets, in the terminal regions of the source proteins (23). This observation underlined the need to apply proper bioinformatics pipelines to filter mass spectrometry (MS) immunopeptidome data prior to making biological interpretations. Finally, the work revealed that the “poor quantity and quality” of most available HLA-DP and DQ immunopeptidome data stemmed primarily from the fact that earlier such datasets were generated using pan–HLA class II antibodies with poor specificity toward these loci (17). This situation changed when anti–HLA-DP and anti–HLA-DQ specific antibodies were applied resulting in peptide yields often comparable to that found for HLA-DR (17, 24, 25).

**Table 1:** *In silico* tools for prediction of peptide binding to HLA molecules.

Tool	Modeling approach	Training data size	Reported performance	Typical use	Key reference
NetMHCIIpan−4.3	Pan−specific artificial neural networks integrating Binding Affinity and Eluted−Ligand MS data with NNAlign_MA	>675,000 peptide–HLA pairs (BA + EL); 142 alleles; MA: SA ~2:1 ratio	ROC-AUCs ~0.95 for DR/DP and ~0.9 for DQ	General−purpose HLA−II binding/presentation prediction for vaccine design, neoepitope ranking, and IEDB workflows	Nilsson et al., Science Advances 2023 (17)
Graph−pMHC	Graph neural network over peptide–HLA complex using Alphafold2−multimer−derived adjacency matrices	>580,000 peptide-HLA pairs (EL); 115 HLA alleles; MA: SA ~4:1 ratio	81.7% absolute average precision	Ranking antibody−drug CD4 T−cell risk regions for de−immunization using pHLA−II presentation scores	Thrift et al., Brief Bioinform 2024 (18)
HLAIIPred	Transformer with cross−attention between peptide and HLA−II pseudo−sequence. Includes new biotherapeutic HLA−II dataset	>595,000 peptide-HLA (EL); 172 HLA alleles; MA: SA ~2:1 ratio	ROC-AUC 0.90 overall; 0.92 for MA data;	*In silico* immunogenicity risk assessment of therapeutic antibodies and neoantigen prioritization	Haghighatlari et al., Commun Biol 2025 (21)
MixMHC2pred	Motif deconvolution of immunopeptidome + position−weight matrix–based pan−allele predictor	>625,000 peptide-HLA (EL); 75 HLA alleles; MA: SA not stated	ROC-AUC 0.95 and 0.85 for IEDB CD4+ epitopes	Pan−allelic CD4 epitope prediction (pathogens, cancer, cross−species alleles) from peptide sequence ± context	Racle et al., Nat Biotechnol 2019 (20); Immunity 2023 (19)

BA, Binding affinity; EL, Eluted ligand; ROC-AUC, area under the receiver operator curve; MA, Multi-allele; SA, single allele ratio; IEDB, Immune Epitope Database.

Graph-pMHC is based on a graph neural network (GNN), which models peptide-protein interactions by representing residues as nodes and their contacts as edges. The network uses an adjacency matrix derived from empirical data and simulations (e.g., Alphafold2) to define the graph edges. The model was trained on 580,292 peptide:genotype pairs from multiallelic data across more than 120 HLA alleles, with non-presented peptides generated from the human proteome (18). The model scans protein sequences to simulate HLA binding by evaluating all potential 9-mer binding core configurations, selecting the correct core during training. The model consists of six stages, including residue embedding, graph enumeration, message passing, and score prediction. An ablation study optimized performance, and the model achieved an absolute average precision of 81.7%. When applied to antibody immunogenicity risk assessment, the model predicted immunogenicity for a database of 100 antibodies with known reported incidences of ADA. Using a threshold of 10% ADA and the model achieved an ROC-AUC of 0.75, indicating its potential for predicting immunogenicity based on peptide presentation data.

HLAIIPred incorporated new data specific for peptides presented from biotherapeutic proteins (21). Using a transformer with cross attention between peptide and HLAII pseudo sequence, with over 595,000 peptide HLA data points and 172 HLA alleles, the model achieved a ROC-AUC of 0.9. Similar to Graph-MHC, when applied to identifying low immunogenicity molecules HLAIIPred performed with an AUC of 0.76. MixMHC2pred is trained on a similar amount of data, >625,000 data points across 75 alleles, achieving an AUC of 0.95 for eluted peptides and 0.85 for peptides identified as CD4+ epitopes in the IEDB (19, 20).

In terms of assessment of protein immunogenicity, the presence of predicted HLA antigen presentation hot spots has been linked to an increased immunogenicity risk (26). Previous concerns of overpredicting peptide binding have largely been resolved with the newer tools trained on MS immunopeptidomics data. Preliminary work has validated predictions of HLA antigen presentation profile compared with MHC-associated peptide proteomics (MAPPs) experiments (27, 28). Specifically, using current *in silico* prediction tools, most MAPPs identified ligands are predicted to be very strong binders (with percentile rank values < 2%, and most often less than 0.5%). The work also underlined the importance of including secondary HLA-DR molecules in the interpretation of immunopeptidome data sets, with a clear example being an identified MAPPs hot-spot region consisting solely of peptides predicted to be HLA-DRB3 restricted (27, 28). These findings give confidence in using *in silico* predictions of HLA antigen presentation in design of biologics and as part of an immunogenicity risk assessment during discovery and preclinical development.

### T cell receptor (TCR) specificity prediction

A current missing link for *in silico* predicted and experimental data for HLA antigen presentation is that these do not include any information related to T cell reactively towards the presented peptides. Given this, the next critical step is to develop tools for prediction of that T cell response, with a lack of data for model development being a major barrier. The peptide-HLA interaction is made up of a constrained interaction of the linear peptide bound to the HLA molecule with rules that are reasonably well understood. The binding of the TCR to the peptide-HLA is a surface-to-surface interaction with more degrees of freedom, suggesting that larger data sets will be needed to understand the rules and therefore to generate predictive models. In addition, the data sets have to represent diversity of TCR repertoire of individuals with a variety of HLA haplotypes, shaped by different immune pre-exposure histories.

Emerging tools aim to predict which T cell receptors (TCRs) could recognise peptide-HLA complexes, though this is not routinely applied in current immunogenicity assessments. Current high throughput TCR sequencing methods can capture representative TCR repertoires, which can be used to investigate the state of the adaptive immune system (29). Past studies have shown that TCR repertoires hold knowledge about past, current and future immune responses (30–32). For the case of immunogenicity, TCR sequencing could enable screening against pre-existing immunity in an individual or a population, as well as unlock novel *in vitro* monitoring solutions. However, TCRs are hyper variable and mostly unique to an individual, necessitating complex analysis protocols. In particular, quantifying T cell immunity with TCR sequencing requires linking the sequenced TCR to their specific targets, which is currently an unsolved problem in the field of immunoinformatics. The most recent public benchmark (IMMREP23) shows that the current state of the art TCR-epitope annotation methods are only able to make accurate predictions for the so-called ‘seen’ epitopes, namely those epitopes for which the used machine learning models have some TCRs to extrapolate from (33). However, for most biologics this will not be the case, as the most likely epitopes derive from the novel sequences. This requires the development of unseen epitope TCR prediction models, which is substantially more complex as it requires correct capturing of the underlying TCR-epitope interaction patterns (34). This may be feasible in the future, however it will require more data.

It is likely that data generated in the adjacent field of neoantigen prediction for cancer immunotherapies will serve the common goal shared by both neoantigen and immunogenicity assessments: identifying strongly immunogenic epitopes. Neoantigen prediction aims to find mutations that create novel non-self epitopes in cancer cells to target with immunotherapies. Recent deep learning (DL) advancements have improved prediction accuracy (35–38), but challenges remain. A study (39) showed that predictions considering T cell responsiveness outperform those based solely on MHC binding. However, a significant failure rate remains, as 95% of predicted neoantigens fail experimental validation due to factors such as immunodominance and limited T cell response training data. There is a need for an unbiased dataset of experimentally validated neoantigens and an open community challenge to improve prediction accuracy in real-world applications.

### B cell epitope predictions

B cell epitope prediction tools lag behind the development of T cell epitope prediction tools, due to factors including the discontinuous nature of B cell epitopes and the ability of B cell receptors (BCRs) to mature. In a case study, predictions from the B cell epitope tool, SEMA (40), for epitopes from human α-galactosidase were validated by experimental mapping, showing consistency with the results from the study (41). For B cell epitope prediction, tools like BipePred 3.0 (42) and SEMA-3D (40) had AUCs around 0.7, indicating reasonable performance in predicting epitopes, with some differences in sensitivities and specificities. Specific risks can be addressed with current tools on the understanding that there is a need for improvement in B cell epitope prediction performance to enable broader application to therapeutic protein development.

## Current predictive capabilities – *In vitro* assays

*In vitro* tools for immunogenicity risk assessment are widely used to understand product related immunogenicity risk and give the opportunity for mitigating risk during the design phase. The risk assessment fulfils the regulatory need at the investigational new drug (IND) stage, although the lack of standardization of assays and guidance currently limit the perceived value of these tools. The industry’s current best practices emphasize a tailored approach driven by the specific attributes of each therapeutic candidate (2). None of the tools individually can predict clinical immunogenicity (1).

**Figure 2:**
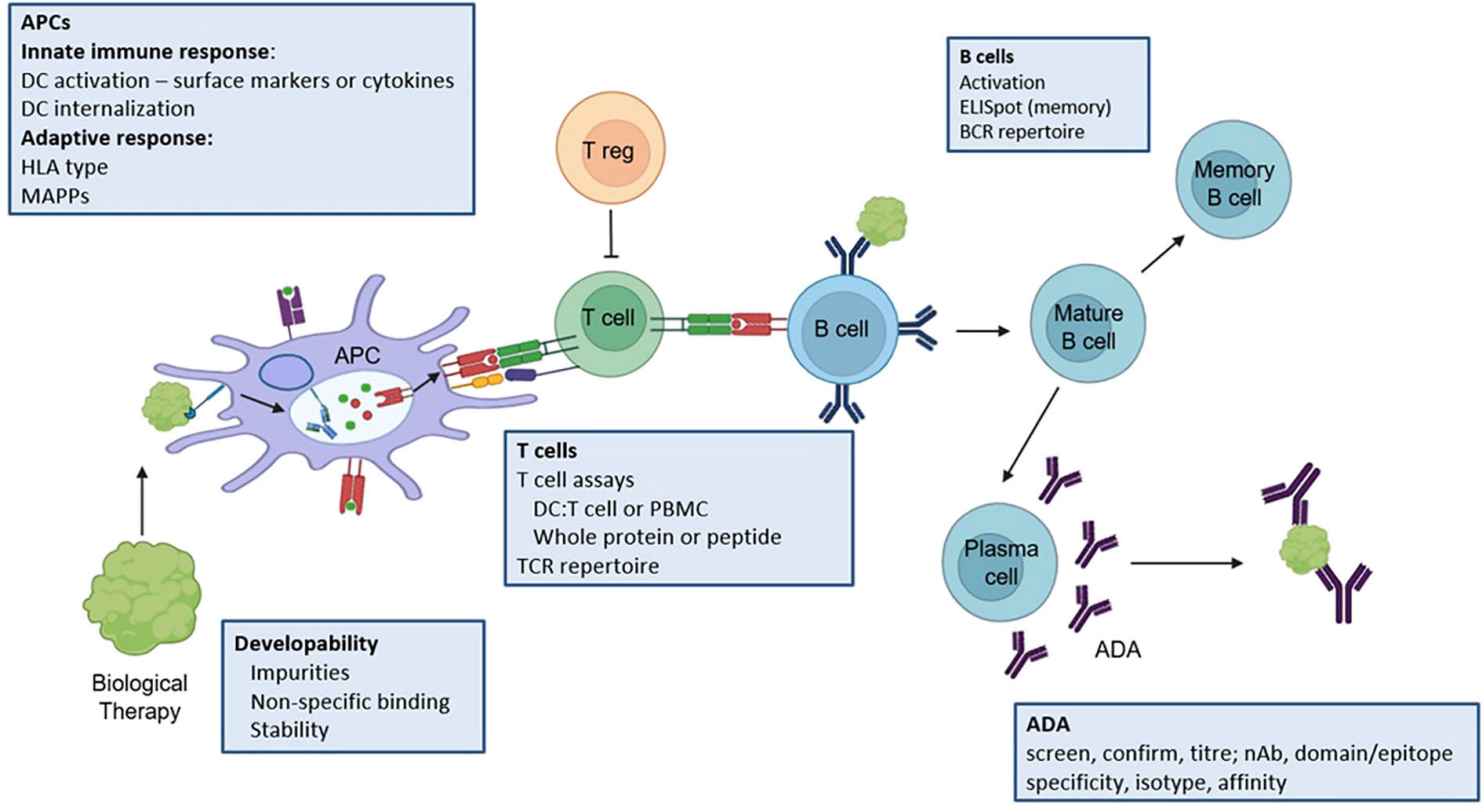
*In vitro* assays contribute to immunogenicity risk assessment during discovery and preclinical development. During clinical trials, ADA measurements and clinical cell samples are used to develop further understanding of the immunogenicity profile and to refine the risk assessment for subsequent trials.

*In silico* tools for identifying potential HLA binding sequences such as those described above and proprietary algorithms such as Epibase^®^ and ISPRI help inform subsequent work to optimize immunogenicity risk. Best practices include using “self”-peptide filters and TCR-facing filters based on the most abundant human proteins as means to model “tolerance”. The pre-clinical *in vitro* immunogenicity assays utilize human-derived peripheral blood mononuclear cells (PBMCs) and help to identify potential immunogenicity concerns early in development and compare immunogenicity across multiple lead candidates. Innate immune response assays address short-term responses to product and process-related impurities. The adaptive immune response assays focus on the longer-term naïve CD4+ T cell response. Examples include MAPPs, which identifies naturally processed HLA binding peptides (43) and CD4+ T cell proliferation assays (44). High-quality PBMCs are essential for reliable assay performance, with different assays employed to address various aspects of the immune response, depending on the target risk and therapeutic modality. Additional *in vitro* tools can be used during clinical development to refine the immunogenicity risk assessment (**Figure 2**).

The bioanalytics of measuring anti-drug antibodies in preclinical and clinical studies consists of a complex series of assays to identify and characterise the ADA. The design and validation of ADA assays is covered by regulatory guidelines (4, 5). The reliability of these assays and the outputs are essential for building immunogenicity risk assessments, although the details of the assays are beyond the scope of this current review.

## Case studies for validation of *in silico* and *in vitro* approaches

### Rare disease and HLA contribution

The pharmacogenomics of immunogenicity describes how genetic factors, particularly HLA types, impact the development of ADAs across various therapeutic areas like hemophilia, TNF inhibitors, and multiple sclerosis (45). In rare diseases with mutations of endogenous proteins leading to insufficient protein activity, replacement with therapeutic proteins usually results in patients being exposed to sequence that their immune system sees as foreign. This lack of tolerance to enzyme replacement factors has been described for the treatment of Pompe’s disease with acid alpha-glucosidase (GAA), for example, with a systematic literature review emphasizing the importance of understanding genetic risk factors for immunogenicity (46).

Polymorphisms in HLA-DRB genes, especially DRB1*15:03 and DRB1*04:05, were associated with higher risks of ADA formation in hemophilia A patients receiving FVIII therapy (47). Over recent years, analysis of ADA data from hemophilia has moved from a statistical analysis to look for data trends and investigate correlations between different variables to a machine learning approach to learn patterns from the data, create learning algorithms and seek to make predictions for new patients. A recent application of this framework to identifying the key biomarkers associated with inhibitors to FVIII identified the top five variables for predicting inhibitor development based on SHAP values are: (i) the baseline activity of the FVIII protein, (ii) mean affinity of all foreign peptides for HLA-DRB3, 4, and 5 alleles, (iii) mean affinity of all foreign peptides for HLA-DRB1 alleles, (iv) the minimum affinity among all foreign peptides for HLA-DRB1 alleles, and (v) FVIII mutation type (48).

### Engineering a low immunogenicity protein

HLA-II and B cell epitope tools have been combined towards engineering a low immunogenicity version of alpha-galactosidase A (AGAL) (41), which is used to treat Fabry disease. A screening approach, termed directed evolution, was used in which amino acids were identified for mutagenesis in site saturation and combinatorial libraries. In addition to identifying mutations that increased protein stability and enzyme activity, the combinatorial libraries were screened with MixMHC2pred to remove putative HLA II binding sequences. The optimised drug candidate, containing 17 mutations, was shown by the MAPPs assay to present fewer peptides overall and fewer clusters than the original drug. A T cell proliferation assay demonstrated a low propensity for activation of T cells. Clinical data for ADA binding to AGAL (49), in combination with the B cell epitope prediction tool, SEMA, was used by Takeda scientists to assess changes in B cell epitope potential of the engineered AGAL. Some small reductions in predicted B cell epitope risk was achieved even though this was not the primary aim of reengineering. Clinical validation data is eagerly awaited.

### Validation of *non-clinical* tools

*In vitro* tools can be applied to new therapies and used for identifying risk factors when ADA emerge in the clinic. A retrospective analysis of the FVII variant Vatreptacog alfa (50) showed where non-clinical immunogenicity assays identified potential risk factors for ADA development. Whilst the native version had 20+ years of clinical use with a well-established profile of safety and efficacy, the new variant with 3 amino acid differences was discontinued in Phase 3 due to safety concerns raised by the presence of ADAs. Application of non-clinical immunogenicity assays identified peptides containing the altered amino acids in *in silico* and *in vitro* assays. Both *in vitro* HLA binding and MAPPs assays identified potential epitopes, which were subsequently shown to activate T cells. The HLA types were consistent between the patients with ADA responses and the ones presenting peptides with high affinity. Additional work to look at how the neo-epitope could be avoided through considering engineering changes on immunogenicity and potency in parallel was performed. Two variants were identified with similar potency to the enhanced FVIIa and with *in vitro* T cell activation like that for the wild type FVIIa (51). Combining the MAPPs assay with T cell activation assays on identified peptides leads to molecules with lower risk, however there are few if any clinical trials making direct comparisons of an immunogenic and deimmunized version of the same molecule.

## Combining *in silico* and *in vitro* for *in vivo* extrapolation of ADA clinical impact

The use of AI in the life sciences has great potential, though risks being limited in impact due to correlation-based predictions missing sufficient detail of the causal pathways of complex biological processes (52). Quantitative systems pharmacology (QSP) models are valuable tools from early discovery to clinical development (53). The QSP framework enables integration of diverse data to predict clinical outcomes and is amenable to development alongside AI advances (54). QSP models of the immune system show promise in simulating immunogenicity outcomes, such as the risk of clinically significant anti-drug antibody (ADA) formation, which can impact drug exposure and efficacy.

**Figure 3:**
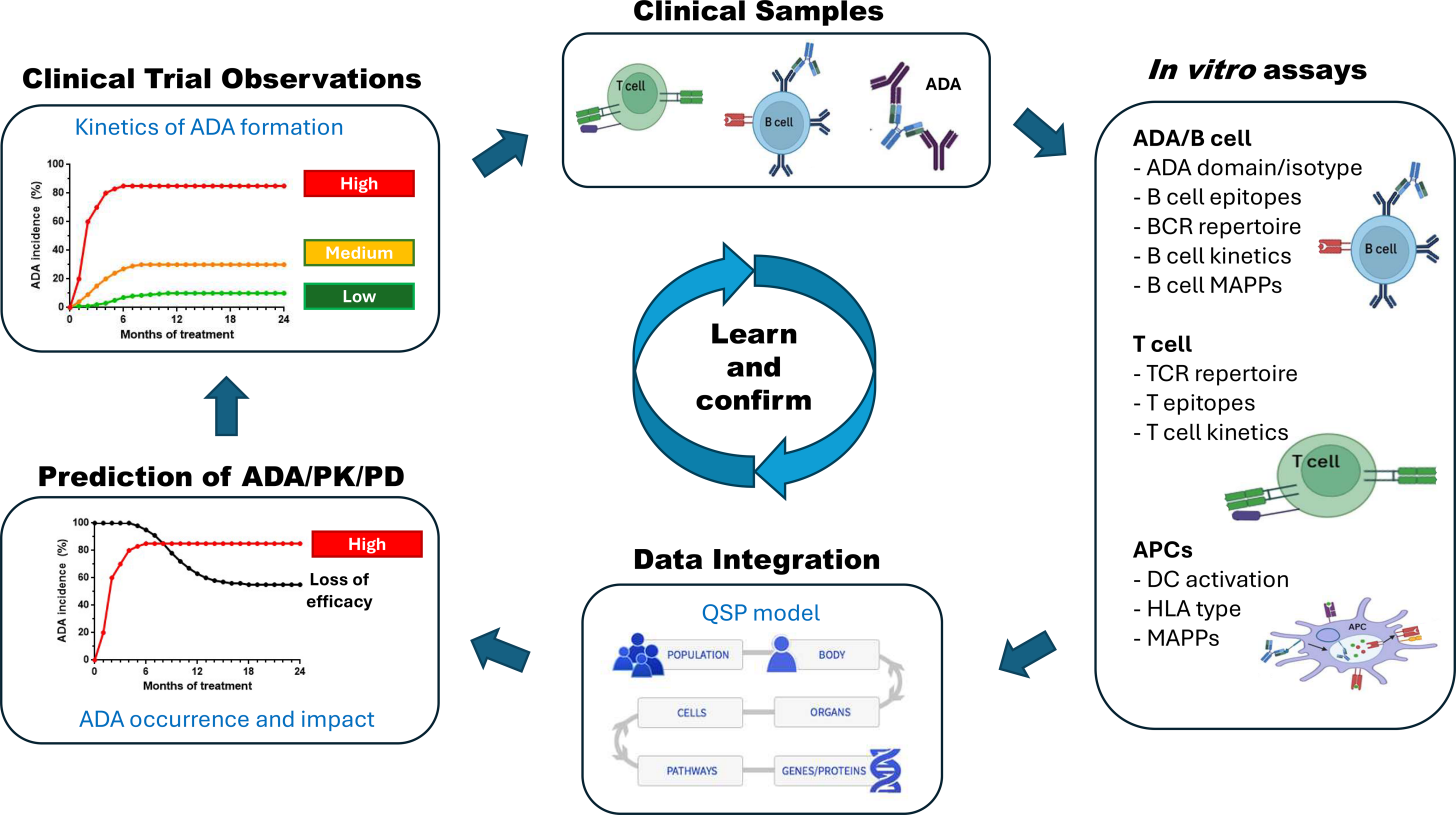
‘Middle-out’ modeling concept, using iterative cycles of learning for immunogenicity simulation. The mechanisms involved in the immune response and the risk factors for generating anti-drug antibodies enable integration of data derived from *in vitro* assays to generate simulations. The outputs of the clinical trials inform subsequent simulations.

Application of QSP approaches to immunogenicity trace their origins to immune system modeling from the 1970s and were further developed with the creation of a fully-integrated immune response model (FIRM) in 2013, a hybrid model that integrated models of multiple immune cell types including dendritic cell, multiple T cell and B cell subtypes and antibody synthesis and immune complex formation (55). The addition of pharmacokinetic (PK) modeling and ADA impacts led to the first working QSP model for simulating immunogenicity and its effect on drug PK (56, 57). The building and validation of QSP models using the “middle out” modelling approach (58), integrating data from AI/ML and experimental methods (bottom-up) with clinical trial observations of the kinetics of ADA formation and impact (top-down) in an iterative learn and confirm cycle to maximize impact for each project (**Figure 3**). These models can be expanded to include emerging mechanisms, e.g. T regulatory cells (59) or different biotherapeutic classes such as gene therapy (60). To avoid overfitting of these models, additional parameters should only be added where sufficient data exists to calibrate them.

Prior to clinical trials, a QSP model can integrate *in silico* tools and *in vitro* assays to simulate risk factors for ADA development (61). In a recent evaluation, the Immunogenicity Simulator was found to predict high vs. low immunogenicity in about 50% of cases, although it tended to overpredict the incidence of ADA (62). The Immunogenicity Simulator was found to correctly predict whether ADA would significantly impact drug PK in 10 of 13 marketed or late-stage antibodies (63). Application of QSP models to a larger set of therapeutic proteins would increase predictive capability, assuming detailed patient level data was available.

These *in silico* models are valuable throughout biologic drug discovery, starting with preclinical immunogenicity risk assessments to guide bioanalytical plans and anticipate adverse events. As clinical data emerges and treatment regimens evolve, QSP models could help assess immunogenicity risks in different patient populations. The framework is also being extended to other therapies, including gene therapies and vaccinations, by incorporating relevant immune mechanisms.

## Industry workflows

Examples of industry workflows were given at the EMBL-EBI workshop from Novartis, MSD, Novo Nordisk and AstraZeneca. The presentations covered various approaches to assessing and mitigating immunogenicity risk in therapeutic proteins, particularly focusing on *in silico* tools and strategies used by companies. A core approach was identified though with differences between each company. This highlights the as yet lack of standards and the opportunity for different models of immunogenicity risk assessment and mitigation to emerge.

**Figure 4:**
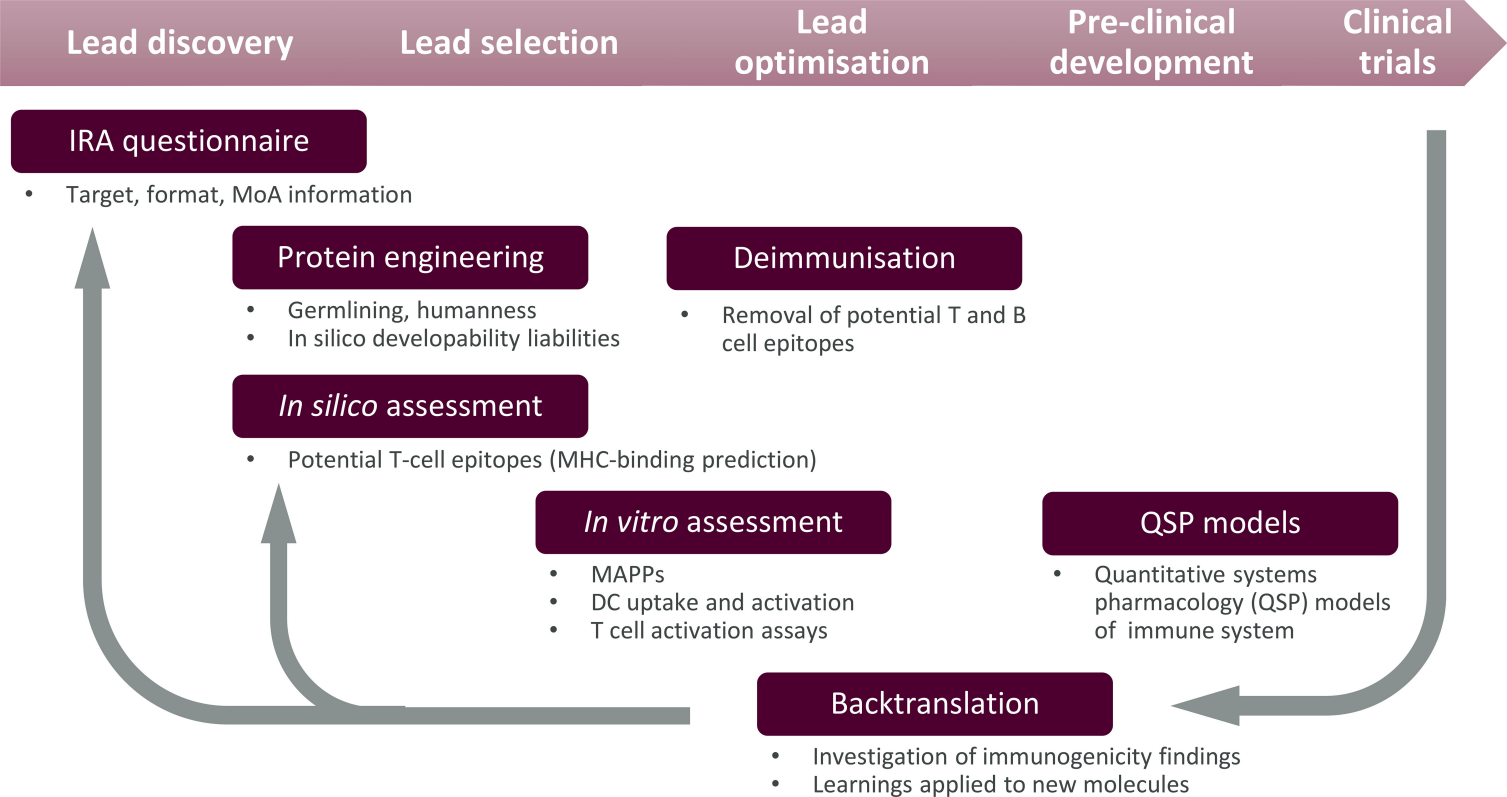
Framework for application of immunogenicity assessment tools during discovery and development of biological therapies. The tools include an immunogenicity risk assessment (IRA) questionnaire, *in silico* and *in vitro* tools. Protein engineering aims for favourable developability profiles and reducing epitope content to optimize the immunogenicity risk level. Less frequently applied tools are the QSP models and clinical sampling to enable backtranslation.

Pharmaceutical companies use *in silico* and *in vitro* tools to assess the immunogenicity of therapeutic proteins (**Figure 4**), particularly in the early stages of drug discovery. *In silico* immunogenicity profiling helps with library screening and lead selection. Based on the *in silico* results and the overall Immunogenicity Risk Assessment (IRA), molecules may then undergo testing in *ex vivo/in vitro* assays. These assays typically fall into three categories: dendritic cell (DC) activation assays, peptide/HLA class II binding and MAPPS, and T cell activation & proliferation assays (**Figure 2**).

In addition to immunogenicity assessment, drug discovery workflows focus on improving the developability and “humanness” of antibody-based therapeutics. By making antibodies more human-like through germlining, the number of potential T cell epitopes can be reduced, lowering the immunogenicity risk. Reducing aggregates and chemical instabilities (like deamidation, isomerization, and oxidation) can also mitigate immunogenicity. For lead optimization, deimmunization strategies are sometimes used to remove potential T and B cell epitopes (64, 65). However, deimmunization must balance removing epitopes with maintaining stability, affinity, and function of the protein. Computational and experimental tools are employed for this process. For example, deimmunization was applied to the anti-factor IXa/X bispecific antibody emicizumab (66), contributing to a low ADA rate (5.1%) in clinical trials.

*In silico* tools primarily rely on HLA binding prediction, a key factor for T cell receptor binding (**Table 2**). Some tools also incorporate antigen presentation data from MAPPS assays, e.g., NetMHCIIpan (67, 68), utilised by Novo Nordisk and AstraZeneca, and iSHAPe, algorithm utilised by Novartis and based on a substantial in-house peptidomics (MAPPs) dataset (69). Other approaches focus on identifying self-peptides, as an exact match and on TCR-facing residues, such self-peptides are less likely to be immunogenic, and peptides that cross-react with common pathogens, which may carry higher risk of immunogenicity, as demonstrated by Novartis during a root cause analysis of adverse events to brolucizumab in the clinic (70, 71).

**Table 2:** Examples of computational tools utilised by pharmaceutical companies for pre-clinical immunogenicity assessment and mitigation.

Company; computational tool name	Basis of the tool	Allele set, binding thresholds	Humanness, similarity to self-proteins, cross-reactivity to pathogens	Correlation with clinical ADA incidence on 217 antibodies set
Novo Nordisk; iPREDICT	Peptide-HLA presentation and binding, based on NetMHCIIpan v3.0-4.3 EL	DR alleles; binders below 5-10% percentile	TCR level self-similarity, humanness scores	r=0.35 (for IRS – immunogenicity risk score) r=0.5 (for inverted humanness scores)
Novartis; iSHAPe	Peptide-HLA presentation and binding, PSSM approach	European DRB1 alleles; population allele frequencies	Higher weight for non-self epitopes (in CDRs); cross-reactive epitopes to pathogens using AP-BLAST (linear ADA epitopes) & MASE (TCR level similarity)	NA
AstraZeneca; ImmunoScreen	Peptide-HLA presentation and binding, NetMHCIIpan 4 EL	27 HLA class II alleles (DR, DQ, DP); binders below 10% percentile; global population allele frequencies	Exact & TCR level self-similarity;	r=0.5 (for total number of epitopes)
MSD; BioPhi	Platform for antibody design and humanisation based on PLM Sapiens	NA	Humanness score OASis	r=-0.53 (for medium OASis identity)

TCR, T cell receptor; iSHAPe, In Silico HLA Aggretope Prediction; PSSM, position-specific scoring matrix; MASE, MAPPs Aggretope Similarity Evaluation; PLM, protein language model.

MSD uses the BioPhi platform (72), which integrates deep learning and language models for antibody humanization, aiming to reduce immunogenicity. The platform includes Sapiens, a deep learning humanization method trained on natural antibody repertoire (73), and OASis, a humanness evaluation score. Their machine learning pipeline also focuses on predicting developability properties, though challenges remain due to small datasets and complex computational features (74). Several computational tools were assessed for their ability to predict ADA incidence in the clinic across 217 therapeutic antibodies that underwent clinical trials (75). Moderate performance was reported, with Pearson’s correlation coefficient r, between 0.35 to 0.53, as shown in **Table 2**.

AstraZeneca employed ML/AI models to predict clinical ADA incidence on the dataset of 260 antibody-based molecules with clinical data. Features included *in silico* T cell epitope content, *in silico* developability properties and additional descriptors like target, indication, route of administration (RoA), and patient cohort immune status. Protein language models (PLM) embeddings (72) were also explored. However, predictive accuracy remained limited (coefficient of determination R² = 0.36), hindered by endpoint noise and missing critical features. AstraZeneca also presented a benchmarking study of *in vitro* assays, namely PBMC and DC:T cell proliferation assays, versus clinically observed immunogenicity on a panel of 10 antibodies. Overall, consensus *in vitro* assays indicated the correct level of immunogenicity risk for 3 out of 9 molecules.

In summary, these companies are leveraging advanced computational tools, such as HLA-binding predictions, humanness scores, and machine learning models, to improve the design of biologics, reduce immunogenicity, and enhance drug safety, though challenges remain in achieving high predictive accuracy, particularly for B cell epitopes and developability predictions.

## Application of risk assessment to new modalities

New therapeutic modalities are emerging that can fundamentally change the course of disease. Cell and gene therapies, including CAR-T, AAV and CRISPR-based gene editing are promising approach for delivering cures to patients (76), with initial breakthroughs in monoallelic rare disease (77). Amongst other developability challenges, each face distinct issues related to unwanted immune responses.

The immune response to viral vector-based gene therapies, such as AAV, both to the capsid and the expressed transgene, can contribute to adverse events and loss of efficacy. During the discovery phase, it is important to focus on risks associated with serotype, vector engineering, target biology, and manufacturability. A key product related risk is the high unmethylated CpG content that has led to increased CTL response and reduced efficacy (78). The activation of innate immunity through TLR and cytokine release can trigger adaptive immune responses with *in vitro* assays currently used to assess the risk. As additional data is generated, ML methods could be developed to help set thresholds of acceptable levels for unmethylated CpG. Emerging approaches to engineer capsids with reduced surface-exposed tyrosine residues (79), reduced T cell epitopes, and other immune evasion strategies using machine learning will progress as additional clinical data becomes available (80). During preclinical development, additional data from biodistribution studies, immunotox assessments, and dose selection further inform the immunogenicity risk assessment. At present the safety data sets are too small to enable ML predicted safety outcomes, though observations of dose dependent complement activation and associated SAEs (78) suggest patterns exist that will be amenable to ML in the future. During the clinical phase, the risk factors are refined and integrated into a comprehensive risk and mitigation plan. Clinical assessments include verifying gene expression in target tissues, monitoring viral DNA in blood, and evaluating immune responses (humoral and cellular). It is anticipated that clinical data will support the future translatability of preclinical studies and risk assessments. The risk of anti-capsid antibodies is mitigated through use of single dosing, although future applications of gene therapy are expected to benefit from repeat dosing.

CAR-T therapies are approved for a range of relapsed or refractory (R/R) haematological malignancies, including B cell acute lymphoblastic leukaemia (B-ALL) in children and adults, diffuse large B cell lymphoma (DLBCL), multiple myeloma, and mantle cell lymphoma. The immunogenicity risks in CAR-T therapies include both humoral and cellular immune responses, necessitating monitoring of these responses during clinical trials. At present there has been no significant impact of immunogenicity on the safety or efficacy of approved CAR-T therapies (81, 82). However, expanding into non-hematological malignancies is expected to increase the immunogenicity risk in addition to other development challenges, with cellular immunity impacting expansion and efficacy in some clinical studies for in development programs. Factors such as the antigen-binding moiety, process-related impurities, and the patient’s immune status all contribute to the immunogenicity risk. Reducing immunogenicity risks through engineering CAR-T cells to minimize epitopes, using the *in silico* and *in vitro* tools discussed above, and applying genome editing techniques to allogeneic CAR-T to evade immune recognition (83, 84). During the clinical phase of development various assays are used to monitor immunogenicity, such as ELISA, ELISPOT, and FluoroSpot for humoral and cellular immune responses.

CRISPR-based gene editing uses the bacterial protein Cas9 to specifically alter DNA sequences, permanently changing a cell’s genetic material. Bacterial proteins are generally immunogenic in humans and use of Cas9 in this system necessitates an understanding of pre-existing and induced T and B cell responses. Identifying a representative array of donors for the pre-clinical cellular assays is an important consideration (85). A combination of T cell assays and MAPPs has been used to demonstrate the likely T cell epitopes for Cas therapies (86), which enables the comparison of alternatives to Cas9 to find those with reduced immunogenicity risk. These modalities may invoke both humoral (ADA) and cellular immune responses, making it crucial to develop pre-clinical assays that reflect these complexities.

Overall, the approach to immunogenicity risk assessment and mitigation in cell and gene therapies builds upon the approach used for other biological therapies, making most use of machine learning in reducing T cell epitope content. Further work to apply ML techniques to B cell epitope reduction and avoid CTL responses is under investigation. Applying AI/ML techniques to clinical outcomes will need additional data from clinical studies.

### Key questions and considerations in immunogenicity prediction

a. Germline as a “protection” against immunogenicity. The role of germline sequences in immune tolerance is a key area of investigation. How the immune system differentiates between “self” and “non-self” peptides and the role of germline immunity in this process remains critical. Further understanding of the various components of the immune pathway, such as the influence of T cell receptors and their sensitivity to non-self sequences, could shed light on how the immune system responds to therapeutic proteins.

b. Allele selection to achieve global population coverage. There is an opportunity to widen the scope of predictive models by incorporating allele data from various populations worldwide, including underrepresented regions like Japan and other parts of Asia. This approach could lead to more globally relevant predictions, increasing the utility of these models in a broader range of patient populations.

c. Self vs. non-self estimation. A key consideration is how to distinguish between “self” and “non-self” peptides. Although we have substantial data on the human genome, the complexity of immune recognition means we may still be lacking information on certain self/non-self interactions. It may only take a few thousand data points to refine this understanding. Companies investigating this issue could share data sets to solve this issue.

d. Single vs. multi-allelic predictions. Current prediction models focus on single allele-driven predictions, i.e. calculating peptide binding one allele at a time. This approach allows for autocorrection during the model’s training process, but it may not fully capture the complexity of the immune system, which involves multiple alleles in each individual. It is an open question as to whether single allele models can be enough or if multiallelic predictions should be prioritized to better represent the immune response in real-world scenarios. Non-clinical data on peptides presented with mono-allelic cell systems (87) in comparison to multi-allelic donors would be a positive step to address this issue.

e. DQ/DP epitope relevance. There’s uncertainty regarding whether DQ and DP epitopes, which bind to HLA class II molecules, are as prevalent in T cell epitopes as DR epitopes. While there are equivalent numbers of binders for these epitopes, their prevalence and relevance in T cell responses to therapeutic proteins remain unclear and require further investigation to understand their significance in immunogenicity assessments. A better understanding of the relative abundance of HLA molecules at the cell surface (88) and deeper investigation of reactive T cells from patients would address this issue.

f. Co-location of T cell and B cell epitopes. T cell and B cell epitopes do not have to be co-located. This was demonstrated in the case of SARS-CoV-2 epitopes in COVID-19 cases (89). In some cases, co-location of epitopes may be a coincidence rather than inevitability. For example, the ‘foreign regions’ of a mAb restricted to the CDRs leads inevitably to the co-location of T and B epitopes.

## Discussion

**Figure 5:**
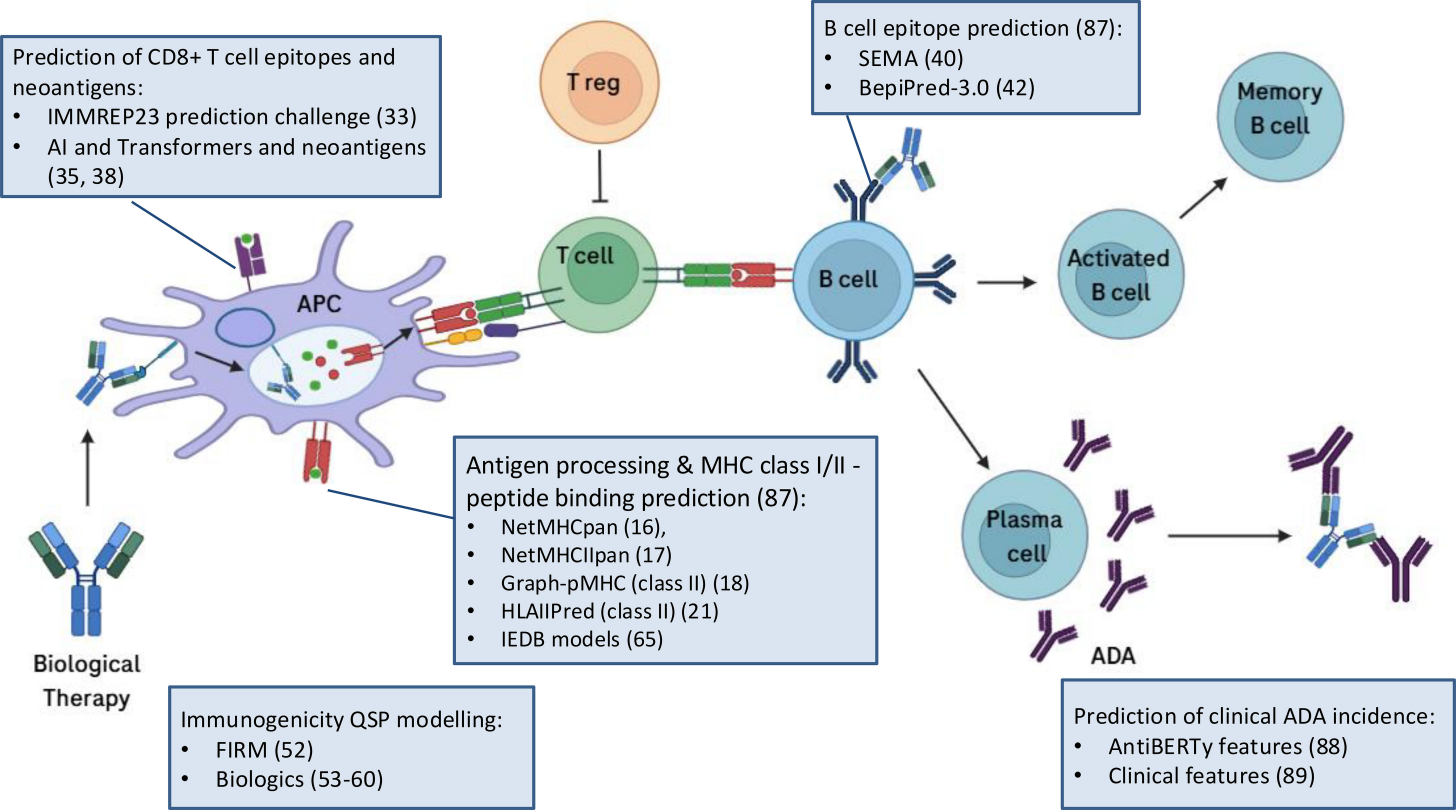
AI/ML tools are used to model, simulate or predict the immunogenicity outcome for biologic therapies. The most frequently modelled and applied process is that of the presentation of peptides on APCs, either in the context of HLA class I or class II presentation. These tools frequently contribute to molecule design or selection during the discovery and preclinical development phases. Tools for predicting B cell epitopes have improved in recent years and can contribute to design or selection of molecules. Holistic prediction of immunogenicity incidence and impact is in the early stages of development.

ML has been used to assist in risk assessment, mitigation and investigation of immunogenicity outcomes for over 20 years. Building on the initial predictions of HLA class II peptide binding, the tools have improved to become more broadly accepted and implemented (**Figure 5**). B cell epitope prediction tools lag behind those for predicting T cell epitopes due to the additional complexity, although they are favoured in some cases. A 2017 review of emerging tools identified the fundamentals needed to predict T and B cell epitopes (90). Today more tools exist for T cell epitope prediction than B cell epitope prediction, yet they still focus on predicting peptide sequences that bind to HLA molecules. Recently, tools for prediction of clinical immunogenicity were developed (91, 92) and achieved moderate success. These models focus on classification of ADA incidence based on a threshold of 2 or 10% and utilise protein language model embeddings (91) and sequence-based features, such as counts of HLA/peptide binders, and additional immunogenicity relevant factors, the MOA, the disease indication and the dose regime (92). Hu et al. (92) demonstrated that these clinical risk factors are important for improvement of ADA incidence prediction, in addition to T cell epitope content prediction. This study utilised internal Roche/Genentech cohort-level clinical studies data and included only ADA assay measurements generated with validated assays.

During the last year, two large cohort-level datasets for clinical ADA data were compiled and published, the Therapeutic Antibody Database (93) and the Immunogenicity Database Collaborative (94), including ADA measurements from around 4300 cohorts covering 300 therapeutics (93) and data from around 1800 cohorts for 220 therapeutics (94). Alongside ADA measurements these databases contain molecule sequences, information on drug format/modality, targets, the drug MoA, disease indication and clinical factors such as treatment regimens, RoA and patient cohort characteristics.

Recently, there has been a rise in multimodal approaches to modelling HLA–peptide binding, T cell epitopes, and clinical immunogenicity. Givechian et al. modelled HLA class I–restricted T cell epitopes by integrating HLA binding data, predicted structures, and biochemical properties of peptide–HLA complexes in the ImmunoStruct tool, with the aim of enabling vaccine and neoantigen discovery (95). TLimmuno2, with a similar focus on vaccines and neoantigens, leveraged transfer learning from a large HLA class II binding dataset to predict T cell responses (96). The modular, multitask T-SCAPE algorithm integrates data and predictions for HLA class I and II binding, TCR–peptide–HLA binding, T cell activation, and humanness to predict clinical ADA incidence (97), reporting 92.5% recall on a published dataset (75) classified using a 10% ADA threshold.

Future development of reliable predictive models for clinical immunogenicity incidence and impact will rely on solving several systematic issues, namely data consistency, data availability, validation of predictive models, and translatability between clinical and non-clinical assays/models.

One of the main challenges is ensuring consistency in the data collected regarding ADA incidences across various studies and platforms. Variability in ADA results can impact the reliability of predictions for immunogenicity, making it crucial to standardize how these incidences are measured and reported. Importance of considering ADA measurements per patient cohort has recently been recognised (92–94), as opposed to averaging ADA values for a molecule across trials with different indications, dosage regimes and routes of administration. Consistency in the outputs of *in vitro* assays, which are commonly used for immunogenicity testing, is another challenge. Differences in experimental protocols, reagent quality, and cell line sources can introduce variability, affecting the interpretation of results and their relevance to real-world immunogenicity. *In vitro* assays must evolve to more accurately reflect the *in vivo* immune response. This includes addressing the limitations of current assays and exploring more relevant cellular models or experimental setups that better simulate human immune reactions in the body. The lack of standardized assays and reference standards for assessing immunogenicity across novel modalities is a significant challenge. There is a pressing need to develop standardized protocols and reference materials that can be widely accepted and implemented across the industry to ensure consistency and reliability. Construction of *in silico* predictive models that are flexible to handle the sources of inconsistencies and work with diverse data types will be key for future efforts. Developing models that can standardize inputs, reduce biases, and produce reliable outputs is essential to creating datasets that can be consistently used in predictive tools.

One of the most promising opportunities for immunogenicity prediction lies in leveraging artificial intelligence (AI), but this comes with challenges related to small data sets. Current databases may not contain enough diverse or large-scale data to make highly accurate predictions in all cases. Proposed advancements in machine learning techniques, like few-shot learning or transfer learning, could help overcome this limitation by training models on smaller, curated datasets that provide high-quality, actionable information. At this time it is not clear how this would be implemented, nor how effective it would be to build models with small data sets. Early examples of this approach, including TCR recognition, show potential, though would need more data to achieve a performance level at which to make drug candidate development decisions (96, 97). Federated model building offers a practical way to a collaborative, privacy-preserving approach that can aggregate data across companies and avoid data privacy and confidentiality limitations. In the case when each company has limited amount of data, the combined dataset may be sufficient to allow for ML/AI model building, benefitting each of the participating companies. The examples of such federated ML are MELLODDY in the small molecule space (98) and FAITE in the antibody developability space (https://faiteconsortium.org/). The prediction of T cell epitopes based on experimental T cell activation/proliferation data is a good candidate for such collaborative approaches.

The accuracy of AI-driven predictions depends heavily on the quality and breadth of the training and test data sets used. Ensuring these datasets are representative of diverse populations, immune responses, and therapeutic modalities is crucial for creating models that generalize well across different conditions and types of drugs. Continuous validation through experimental data will be necessary to assess the real-world utility of these models. There is a need to better understand the clinical relevance of immunogenicity predictions and their correlation with real-world outcomes. This includes addressing whether *in silico* predictions correlate with observed clinical immunogenicity in patients and what factors might influence these outcomes. It is crucial to evaluate the need for using multiple prediction tools versus identifying the most relevant ones. While using a broad array of tools may provide insights into different aspects of immunogenicity, there may be advantages to focusing on the most precise and relevant models, reducing complexity and improving prediction accuracy. *In silico* tool development beyond HLA-peptide binding predictions to develop more advanced mathematical models and model-informed drug development (MIDD) approaches could provide more actionable insights into immunogenicity. These approaches could help integrate data from multiple sources and predict not only the risk of immunogenicity but also the potential therapeutic outcomes of new drugs.

Regarding translation from clinical findings to non-clinical assays and models, there is value in reviewing drugs that fail during clinical development due to ADA, which are usually presented in overview at meetings and symposia, yet not frequently published in enough detail to help the field develop. Questions that could be answered to help are: how could this have been detected earlier and avoided? And what were the mechanisms contributing to ADA formation and subsequent impact? To provide information which could improve computational tools, additional experimental investigation of patient samples is required here, e.g. confirmation of T cell epitopes using PBMCs from ADA positive individuals, or HLA-typing patients cohorts (71, 99).

In summary, the challenges in immunogenicity prediction are multifaceted, requiring careful consideration of data consistency, model development, and the integration of new technologies like AI. There are clear opportunities to improve our understanding and prediction of immune responses, but substantial work remains in standardizing methods, refining prediction models, and addressing the clinical relevance of predictions.
